# Diagnostic accuracy of photodynamic diagnosis with 5-aminolevulinic acid, hexaminolevulinate and narrow band imaging for non-muscle invasive bladder cancer

**DOI:** 10.7150/jca.34527

**Published:** 2020-01-01

**Authors:** Changhao Chen, Hao Huang, Yue Zhao, Hao Liu, Yuming Luo, Richard J. Sylvester, Jia ping Li, Thomas B. Lam, Tianxin Lin, Jian Huang

**Affiliations:** 1 Department of Urology,; 2 Guangdong Provincial Key Laboratory of Malignant Tumor Epigenetics and Gene Regulation, Sun Yat-sen Memorial Hospital, Sun Yat-sen University, Guangdong, P. R. China; 3 Department of Interventional Oncology, Sun Yat-Sen University First Affiliated Hospital, Guangzhou, China;; 4 Department of Urology, Chengdu Fifth People's Hospital, Chengdu, P. R. China; 5 Department of Pancreatobiliary Surgery, Sun Yat-Sen Memorial Hospital, Guangzhou, Guangdong P. R. China; 6 European Association of Urology Guidelines Office, Arnhem, The Netherlands; 7 Academic Urology Unit, University of Aberdeen, Aberdeen, UK

**Keywords:** non-muscle-invasive bladder cancer, diagnostic accuracy, narrow band imaging, photodynamic diagnosis, white light-guided cystoscopy

## Abstract

**Objective:** To assess the diagnostic test accuracy (DTA) of photodynamic diagnosis with 5-aminolaevulinic acid (5-ALA), hexylaminolevulinate (HAL) and narrow band imaging (NBI) for non-muscle-invasive bladder cancer (NMIBC), with white light-guided cystoscopy (WLC) as reference standard.

**Materials and Methods:** A systematic review and narrative synthesis was performed in accordance with PRISMA. Major electronic databases were searched until 20^th^ May 2019. All studies assessing the DTA of 5-ALA, HAL and NBI compared with WLC at patient and lesion-level were included. Relevant sensitivity analyses and risk of bias (RoB) assessment were undertaken.

**Results:** 26 studies recruiting 3979 patients were eligible for inclusion. For patient-level analysis, NBI appeared to be the best (median sensitivity (SSY) 100%, median specificity (SPY) 68.45%, median positive predictive value (PPV) 90.75%, median negative predictive value (NPV) 100% and median false positive rate (FPR) 31.55%), showing better DTA outcomes than either HAL or 5-ALA. For lesion-level analysis, median SSY across NBI, HAL and 5-ALA were 93.08% (IQR 87.04-98.81%), 93.16% (IQR 91.48-97.04%) and 94.42% (IQR 82.37-95.73%) respectively. As for FPR, median values for NBI, HAL and 5-ALA were 20.40% (IQR 13.68-27.36%), 17.43% (IQR 12.79-22.40%) and 28.12% (IQR 22.08-42.39%), respectively. Sensitivity analyses based on studies with low to moderate RoB and studies with n>100 patients show similar findings.

**Conclusions:** NBI appears to outperform 5-ALA and HAL in terms of diagnostic accuracy. All three modalities present high FPR, hence indicating the ability to detect additional cases and lesions beyond WLC.

## Introduction

Bladder cancer is one of the most frequently diagnosed tumor types, with an estimated 166,583 newly diagnosed cases and 58,742 deaths due to the disease in Europe in 2012, about 75% among which present as non-muscle invasive bladder cancer (NMIBC) 1-4. Most patients with NMIBC develop a recurrence (up to 70%) within 5 years after initial treatment 5-7. Traditional white light (WL) guided transurethral resection of bladder tumors (TURBT) has been regarded as the gold standard method for diagnosis and treatment. However, the accuracy of white light cystoscopy (WLC) in detecting disease is unsatisfactory, which leads to residual untreated disease or missed coexisting carcinoma in situ (CIS), mainly due to overlooked lesions. Recurrence after TURBT is remarkably common with up to 30% of patients having tumor identified at the first-check cystoscopy at 3 months and 50% of patients developing a recurrence within the first year 8, 9. Thus, new imaging technologies are being developed to improve visualization of tumors, which can assist urologists in achieving complete resection and reducing the risk of recurrence.

Photodynamic diagnosis (PDD) is applied with intravesical instillation of 5-aminolevulinic acid (5-ALA) or hexaminolevulinic acid (HAL) under blue-violet (380-440nm) light. The effect of 5-ALA on tumor detection in the urinary bladder has been confirmed in several clinical trials 10-12. 5-ALA guided cystoscopy has been shown to be an efficient method of mapping the entire mucosa to detect urothelial tumors and flat CIS lesions^13^. HAL is the lipophilic hexylester of 5-ALA and has been commercially available since 2006, which is considered an efficient diagnostic tool in the detection of NMIBC. However, recent prospective, randomized studies have challenged the benefits of PDD^14^.

Narrow band imaging (NBI) is an image-processing modality filtering WLC to two narrow band widths of 415 and 540 nm, which corresponds to the visible blue and green light spectra, respectively. In contrast to PDD, NBI does not require fluorescent agents to improve the visualization of vascularized mucosal lesions, such as NMIBC. The utility of NBI for increasing tumor detection compared with WLC has been confirmed in several initial trials 15-17. Though early results are encouraging, there is currently limited experience with NBI in detecting bladder cancer. NBI may also result in increased false-positives, especially for patients with prior intravesical instillations 18, 19. The specific objective of our study was to perform a systematic review assessing the diagnostic accuracy of PDD using 5-ALA, HAL, and NBI against the reference standard of WLC for NMIBC.

## Materials and Methods

### Literature-search strategy

The review was performed according to Preferred Reporting Items for Systematic Reviews (PRISMA)^20^ and Standards for Reporting Diagnostic Accuracy Studies (STARD)^21^. Databases including PubMed/MEDLINE, PMC, Web of Science, the Cochrane Library, Central Register of Controlled Trials and Embase were systematically searched from inception up to 13^th^ May 2019, using the following MeSH and combined terms which were adjusted for the different databases: “photodynamic diagnosis, PDD, hexaminolevulinate, HAL, 5-aminolevulinate acid, 5-ALA, narrow band imaging, NBI, white light cystoscopy, bladder cancer, bladder tumor and BCa.” The search was supplemented by additional sources including the reference lists of all included studies. Only full text articles published in the English language were included. At least two reviewers (CC and HL) screened all abstracts and full-text articles independently. Disagreement was resolved by discussion or by reference to an independent arbiter (JH). Exclusion criteria were animal studies, reviews, historical overviews, or editorials. For missing or unclear data, we contacted the authors to get more information.

### Inclusion and exclusion criteria

All prospective and retrospective studies reporting the diagnostic accuracy of photodynamic diagnosis (PDD) with 5-aminolevulinic acid (5-ALA), hexaminolevulinate (HAL), or narrow band imaging (NBI), with WLC as reference standard, were included. Additional inclusion criteria included the following elements: 1) Population: Patients aged ≥18 years with suspected NMIBC in the primary setting (i.e. primary diagnosis), or patients with previously confirmed NMIBC undergoing surveillance (i.e. diagnosis of recurrent tumours). Previous intravesical chemotherapy instillation was not described. NMIBC included Ta, T1 and CIS; 2) Reference standard: All patients must have had WLC as the reference standard, with positive or negative cases being denoted by the presence or absence of NMIBC confirmed by histopathological examination; 3) Diagnostic performance should be compared in intra-patient groups. 4) Outcomes: The primary outcomes were sensitivity (SSY), specificity (SPY), positive predictive value (PPV), negative predictive value (NPV), false positive rate (FPR), and false negative rate (FNR). If the outcomes were not reported in the study, these were derived and calculated from the available data based on the construction of 2x2 tables.

We limited these criteria to studies published in the English language and to original studies only. When two or more studies reported on a group of patients at the same institution during an overlapping time period, only the article with the latest data set was included, unless different outcomes were reported or different subgroup analyses were performed.

### Data Extraction

Data from included studies were extracted by two independent authors (YZ and CC). A data extraction form was developed to collect information, including the first author, study characteristics (country, study design, number of patients, study end points, follow-up), intervention characteristics (index tests, reference standard, duration of follow-up, schedule and nature of WLC), patient characteristics (age, sex, NMIBC patients, biopsy lesions, tumor lesions, disease grade and stage, disease setting), and diagnostic accuracy measure as previously specified. Any unresolved discrepancies were resolved by consensus or referred to an adjudicating senior author (JH).

### Quality assessment strategy

The Quality Assessment of Diagnostic Studies-2 (QUADAS-2) 22 was performed on included studies. The risk of bias (RoB) was scored as “yes,” “no,” or “unclear” for each domain to designate a low, high, or unclear RoB, respectively. The scoring was performed independently by two authors (YZ and CC); disagreement was resolved by discussion or with an independent arbiter (JH). We arbitrarily defined “low RoB” as at least 3 domains scoring “low” across both categories without any domains scoring “high” across either category; “moderate RoB” as at least 2 domains scoring “low” across both categories and without any domain scoring “high” across either category; all other scoring patterns were defined as “high” RoB.

### Statistical analysis

Data was extracted from each study at lesion or patient level to assess 5-ALA, HAL and NBI as the index test using WLC as reference standard, with positive or negative disease as determined by histopathological examination. 2×2 tables were used to summarize the above data. These tables were used to calculate the primary outcomes of SSY, SPY, NPV, PPV, FPR and FNR. Studies reporting insufficient data were excluded. SSY was defined as the proportion of index test-positive patients or lesions out of all cases of WLC-positive findings. SPY referred to the proportion of index test-negative patients or lesions out of all cases of WLC-negative findings. NPV was defined as the proportion of true negatives (i.e. negative index test and negative WLC) out of all index test-negative cases or lesions; PPV was defined as the proportion of true positives (i.e. positive index test and positive WLC) out of all index test-positive cases or lesions. FNR was defined as the proportion of index test-negative cases or lesions out of all cases of WLC-positive findings; FPR was defined as the proportion of index test-positive cases or lesions out of all cases of WLC-negative findings. FPR provides measurement of additional diagnostic value of PDD or NBI over WLC, as FP cases or lesions referred to patients who had index test-positive findings whilst WLC found negative findings. Because of the expected clinical and methodological heterogeneity across studies, only a narrative synthesis was performed. All diagnostic test accuracy (DTA) outcomes were presented as proportions (%) for individual studies and summarized as median and interquartile range (IQR) for all studies collectively. The pooled estimates for Hierarchical Summary Receiver Operating Curve (HSROC) with 95% confidence intervals (CIs) of the compared end points were used, which is an overall summary measure index of the diagnostic accuracy. A perfect test will have an Area Under Curve (AUC) close to 1 and a poor test has AUC close to 0.5. Results were plotted on HSROC using Stata 13.0 (StataCorp, College Station, TX, USA). To explore the effect of heterogeneity on the results, sensitivity analyses were planned based on patient-level vs lesion-level analysis, disease grade (low grade vs high grade), stage (pTa vs pT1), setting (primary vs recurrent tumours), number of participants (studies with n>100 patients only), and on studies with low to moderate RoB.

## Results

### Quantity of evidence identified

**Figure 1:**
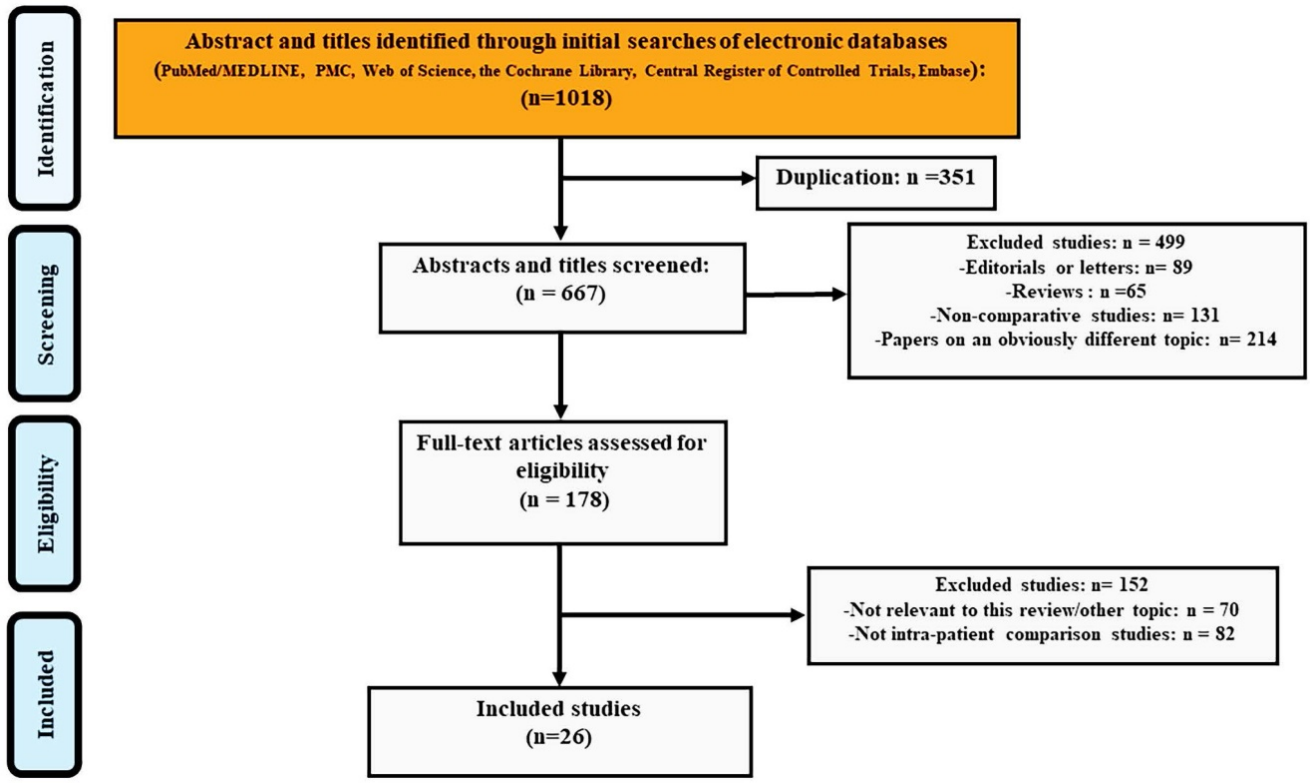
The PRISMA flow chart of included studies in DTA analysis.

Figure 1 showed the number of citations retrieved and the selection flow diagram for the studies included in the analysis. The search yielded 1018 entries and 351 of these were duplicates. We excluded 499 studies when screening titles and abstracts: 89 editorials or letters, 65 reviews or meeting abstracts, 131 non-comparative studies and 214 papers on an obviously different topic. During the screening of 178 full-text articles, 70 studies were excluded for not being relevant to this review and another 82 studies were excluded for not having within-patient comparisons. Finally, 26 studies^17^, ^23^-^47^ were included in the DTA analysis.

### Characteristics of the included studies

Table 1 summarized the baseline characteristics of the included studies. The 26 included studies enrolled 3979 BC patients (Table 1). The interventions were 5-ALA-based PDD in 9 studies, HAL-based PDD in 8 studies, and NBI in 9 studies. The studies were published from 1994 to 2016, and the sample size ranged from 12 to 605 participants, with a median sample size of 95.5. The mean or median age in the studies was quite similar. Likewise, the male/female ratio showed no differences. Most enrolled patients in included studies were NMIBC (90-100%), while only a few studies described the number of patients according to disease setting (i.e. primary vs recurrent tumors) (Table 2).

### Diagnostic test accuracy results

Table 3 showed the results of individual studies included in the DTA analysis. All studies used non-standardized definitions to calculate their DTA outcomes, in which case the results were recalculated using standard definitions with the raw data provided (Table 3). DTA results are presented for all included studies based on patient-level and lesion-level analyses. In the patient-level analysis, the median sensitivity for NBI, HAL and 5-ALA were 100% (IQR 100-100%), 100% (IQR 91.67-100%) and 100% (IQR 89.67-100%), respectively. NBI appeared to be marginally the best with slightly higher quartile values. Median specificity for NBI, HAL and 5-ALA were 68.45% (IQR 39.57-96.47%), 41.18% (IQR 33.09- 66.97%), 58.91% (IQR 57.23-75.04%) respectively, with NBI presenting a higher specificity. NBI also showed highest values for PPV (90.75%, IQR 82-95.59%) and NPV (100%, IQR 100-100%). The median FNRs for NBI, HAL and 5-ALA were 0 (IQR 0-0), 8.33% (IQR 4.17-12.5%) and 0 (IQR 0-10.33%) respectively, with NBI appearing to be the best with the lowest FNR. The median false positive rate for NBI, HAL and 5-ALA were 31.55% (IQR 3.54-60.08%), 58.82% (IQR 33.03- 66.91%) and 41.09% (IQR 24.96-42.77%) (Table 4) respectively, showing the ability to detect additional cases beyond WLC. Moreover, The HSROC for NBI, HAL and 5-ALA were showed in Figure 3, supplementary figure 1 and supplementary figure 2, the AUC of NBI, HAL and 5-ALA were 0.91 (95% CI, 0.88- 0.93), 0.94 (95% CI, 0.92-0.96) and 0.82 (95% CI, 0.79- 0.85), presenting excellent diagnostic performance compared with WLC. In this regard, HAL-based PDD appeared to provide the greatest value in detecting additional cases. However, overall, NBI showed better diagnostic accuracy outcomes than HAL or 5-ALA, with greater sensitivity, PPV and NPV.

Moreover, we evaluated the diagnostic accuracy outcomes of NBI, HAL and 5-ALA based on lesion-level analyses, which was showed in Table 4, assessing the diagnostic efficacy of NBI, HAL and 5-ALA on suspicious lesions. The median sensitivity for NBI, HAL and 5-ALA were 93.08% (IQR 87.04-98.81%), 93.16% (IQR 91.48-97.04%) and 94.42% (IQR 82.37-95.73%) respectively, with these methods showing similar outcomes. Median specificity for NBI, HAL and 5-ALA were 79.60% (IQR 72.64- 86.32%), 82.57% (IQR 77.60-87.21%), 71.88% (IQR 57.61-77.92%) respectively, with HAL having a highest value; NBI showed the highest PPV (76.59%, IQR 65.60-81.76%) and 5-ALA the highest NPV (100%, IQR 100-100%). The median FNRs for NBI, HAL and 5-ALA were 6.92% (IQR 1.19-12.96), 5% (IQR 1.49-7.65%) and 5.58% (IQR 4.27-17.63%) respectively. HAL appeared to be best with the lowest FNR. The false positive rates for NBI, HAL and 5-ALA were 20.40% (IQR 13.68-27.36%), 17.43% (IQR 12.79-22.40%) and 28.12% (IQR 22.08-42.39%) respectively. NBI, HAL and 5-ALA appeared to be efficient diagnostic methods for lesion detection.

### Sensitivity analysis

WLC was used as standard reference in all the included studies. WLC protocol was similar among included studies and applied on suspicious lesions. SSY and FPR could provide a measure of diagnostic value of PDD or NBI over WLC. When we excluded studies with high RoBs, it showed that NBI had a higher sensitivity (95.85%, IQR 88.80-99.60%) and FPR (25.01%, IQR 19.02-28.34%) than HAL or 5-ALA at lesion-level (Table 5, Supplementary Table 1-3). For studies with low to moderate RoB at patient level (Table 5), 5 studies (i.e. 1 study for 5-ALA, 1 study for HAL and 3 studies for NBI) were included. The analysis showed that NBI had a higher sensitivity (100%) and FPR (59.09%) than HAL or 5-ALA. For sensitivity analysis based only on studies recruiting at least 100 patients for patient-level and lesion-level analysis (Table 6), NBI had a higher sensitivity (100%) and FPR (33.53%) than HAL or 5-ALA at patient-level analysis; at lesion-level analysis, NBI was also found to have higher sensitivity (92.86%, IQR 88.69-97.3%) than HAL (93.16%, IQR 91.48-97.04). No data were available on grade and stage of disease, primary vs recurrent disease, and duration of follow-up, to enable additional sensitivity analyses.

### RoB of included studies

**Figure 2:**
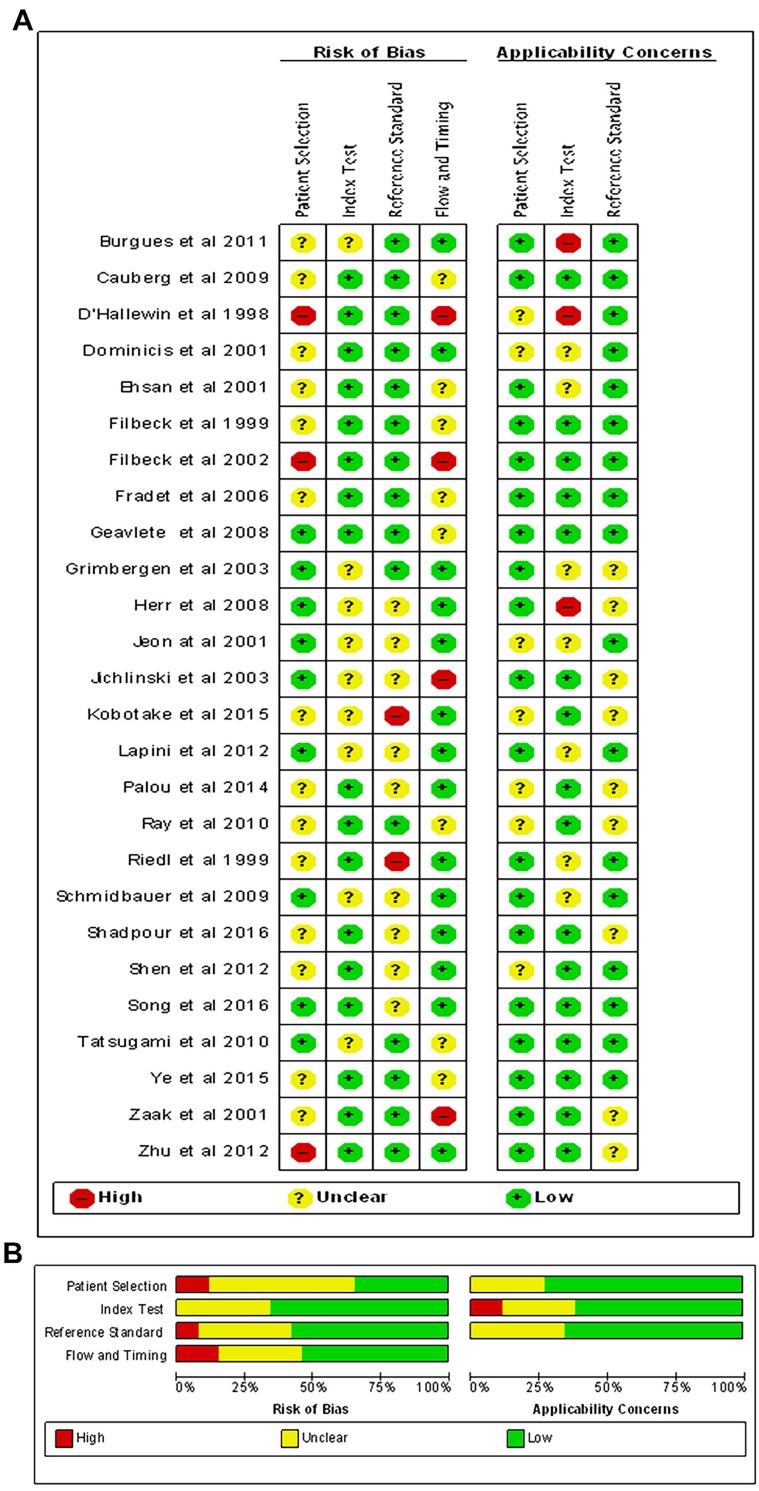
Quality assessment of included studies. The Overall (A) and Study-level distribution plot for risk of bias using QUADAS-2 tool. Studies are deemed to be at high, low or unclear risk of bias for each domain. The review authors' judgments about each domain are presented as percentages across all included studies.

**Figure 3:**
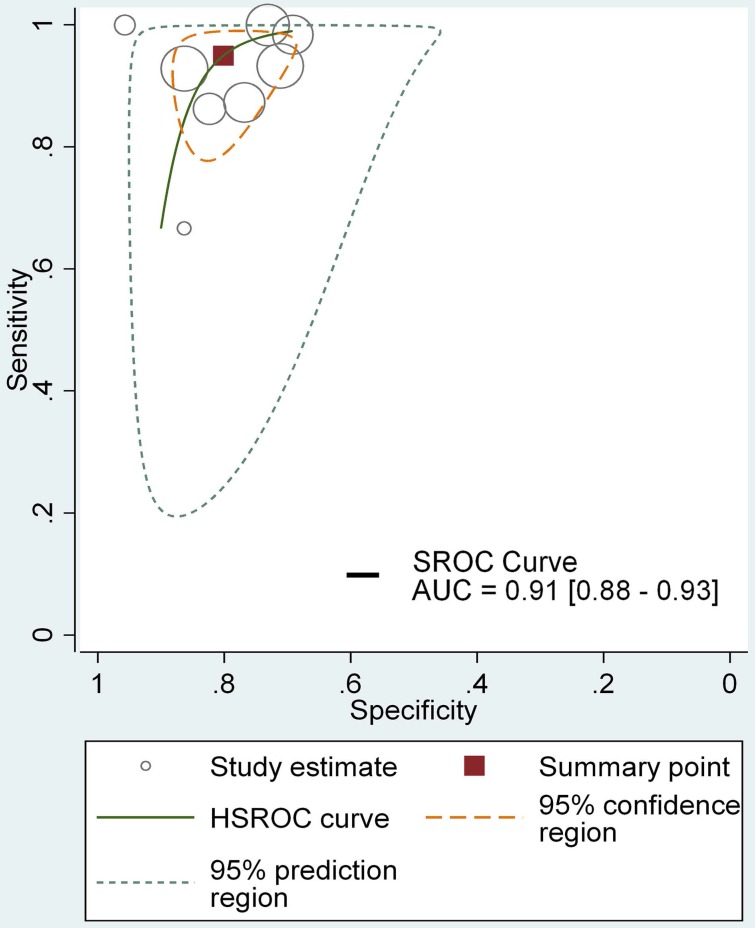
The HSROC curve for NBI diagnosing NMIBC comparing with WLC in lesion level.

Figure 2 showed the RoBs assessment for studies included in the review using the QUADAS-2 tool. Overall, most studies (18/26) were judged as having low or unclear RoB across most domains. While patient selection was generally acceptable in the studies included, a few studies did not clearly report the inclusion criteria. All studies clearly reported methodology for the index test and reference standard, and were not considered a significant source of potential bias. Moreover, several studies were at high RoBs for the flow and timing, as many included targeted biopsies of suspicious lesions. These studies were excluded from diagnostic analyses because the FN data were not accurate.

## Discussion

Firstly, based on data from 26 studies, our systematic review found that almost all NMIBC diagnosed by WLC could be detected with PDD using 5-ALA, HAL or NBI. Second, the median rate of NBI or PDD-detected NMIBC outside WLC was positive (i.e. FPR >0), which indicates that NBI or PDD showed addition diagnostic benefits over WLC. Third, NBI could diagnose more NMIBC lesions than PDD-based HAL or 5-ALA-guided cystoscopies. Taken together, our results suggest that NBI could potentially be the best diagnostic intervention for NMIBC patients.

Intuitively, detection of more lesions and efficient treatment should lead to a better prognosis. In the present review, we have summarized the diagnostic accuracy of the new imaging-based diagnostic strategies for NMIBC. Our results indicate that the diagnostic accuracy of both PDD and NBI-guided cystoscopy were better than WLC. Moreover, we noted that NBI and PDD showed addition diagnostic benefits over WLC for NMIBC patients which would otherwise have been missed by conventional WLC. NBI was associated with the highest FP rate at lesion level, which suggests it play important role in minimizing missed lesions. Although data at patient level may be more relevant clinically, most of the included studies reported data at lesion level only. These findings demonstrate a numerical consistency, although a meta-analysis was not undertaken. In addition, whether these technologies should be applied to replace WLC or to augment WLC remains unclear. Since virtually all of the techniques assessed in this review had median sensitivities of 100% based on the reference standard of WLC, the review findings suggest that they are at least as good as WLC in detecting NMIBC, and are likely to surpass WLC in terms of diagnostic accuracy because of additional benefit of detecting more cases at patient-level and lesions at lesion-level. In this context, there are compelling reasons to adopt these strategies in clinical practice, although their clinical effectiveness in terms of important oncological outcomes such as progression and survival, or cost effectiveness, remains unproven. These present findings are not sufficient to trigger a change in clinical practice, while they do strongly suggest that new imaging-based technologies, in particular NBI, are promising and deserve further assessment through well-designed, protocol-driven prospective studies using standardized definitions and reference standards with robust follow-up protocols. The European Association of Urology guidelines (2018) acknowledge the improved detection rate of fluorescence-guided cystoscopy for malignant tumours, particularly CIS^48^. Future studies should investigate if the intervals of surveillance cystoscopies could be altered when PDD or NBI is used, and the necessity of re-TUR after PDD and NBI should be evaluated.

The most sensitive interventional diagnostic strategy for NMIBC patients remains unclear. Our study supports the assertion that new imaging-based techniques should be recommended. A high FPR reflects the inadequacy of WLC as a diagnostic procedure and a better detection of NMIBC with PDD or NBI compared with WLC. Although this appears counter intuitive (i.e. how can a histologically proven diagnosis of NMIBC based on an index test be classed as a 'false positive'), this is because all diagnostic accuracy measures must be calculated based on the results of the reference standard (i.e. WLC) as denominator. Consequently, a high FPR reflects the sub-optimal performance of WLC in diagnosing NMIBC, and better performance of the new imaging technologies.

PDD and NBI both aim at improving the visualization of bladder tumors. Randomized studies 17, 49, 50 have shown the superiority of PDD or NBI over WLC alone in tumor detection. SSY for PDD ranges from 76% to 97% compared with 46-80% for WLC. A random-effect meta-analysis using 2807 patients from 27 studies found a 21% increase in tumor detection with PDD over WLC in the pooled estimates for patients and biopsies^13^. NBI, another optical enhancement technology, increases the contrast between vasculature and superficial tissue structures of the mucosa by excluding the red spectrum of WLC. Several studies previously reported the enhanced diagnosis of bladder tumors with NBI cystoscopy compared with standard WLC alone 17, 18. Our previous meta-analysis indicated that NBI provides comparable or higher diagnostic precision than WLC and an additional 17% of patients and an additional 24% of tumors were detected by NBI 51. However, these studies did not use standardized diagnostic test accuracy definitions.

The previous review by Karaolides et al^52^ reported that both PDD using 5-ALA and HAL improves tumour detection after TURBT compared with WLC. Burger et al^53^ reported that PDD using HAL significantly improves the detection of bladder tumours leading to a reduction of recurrence at 9-12 months. Moreover, Li et al indicated that NBI provides comparable or higher diagnostic precision than WLC 51 and the other meta-analysis^54^ evaluated WLC, PDD- and NBI-assisted TUR in the NMIBC patients, suggesting both PDD and NBI for NMIBC were superior to WLC in lowering the recurrence rate. Therefore, although a clear benefit for PDD and NBI have been found for improving the detection of NMIBC, the best diagnostic strategy remains controversial. The findings support that NBI might be the most sensitive interventional treatment for NMIBC patients.

The strengths of our study include the stringent methodology used to synthesize the evidence, including adhering to PRISMA guidelines, using standardized definitions of diagnostic test accuracy, undertaking a systematic and comprehensive search strategy, and utilizing explicit inclusion and exclusion criteria in relation to design of primary studies, population, index tests, reference standard and outcomes. Quality assessment was also performed using QUADAS-2. However, potential study limitations should be acknowledged. Firstly, any biases and inaccuracies within individual studies would be reflected in our analysis. The greatest threat to the validity of the study is heterogeneity in study designs, recruitment criteria, interventions, or endpoint assessments. The lack of data on key clinical variables may also introduce clinical heterogeneity, including grade and stage of disease, duration of follow-up, and primary vs recurrent disease, prevented further sensitivity analyses. Also, we could not explore the diagnostic performance of PDD and NBI in tumour recurrence or intravesical instillation settings compared with WLC due to lack of data. However, we have attempted to minimize biases by applying rigorous selection criteria during the design phase of our study, standardizing data extraction and performing several sensitivity analyses to evaluate the robustness of our findings.

In summary, NBI appears to be the most sensitive diagnostic intervention for NMIBC patients compared with either HAL or 5-ALA, both of which are PDD-based, based on diagnostic test accuracy assessment using WLC as a reference standard. NBI, HAL and 5-ALA all demonstrated median sensitivities of 100%, and appeared to have the ability to detect additional cases of NMIBC both at patient and lesion levels. Sensitivity analysis suggests that NBI can diagnose more additional NMIBC lesions compared with either HAL or 5-ALA. The findings confirm the excellent diagnostic performance of these new imaging-based technologies in diagnosing NMIBC in comparison with the present standard using WLC, although well-designed prospective studies with long-term follow-up may shed more light on their impact on key oncological outcomes such as progression and survival.

## Supplementary Material

Supplementary figures and tables.

## Figures and Tables

**Table 1:** Summary of the characteristics of the included studies

Study	Country (No. of institutions)	No. of patients	Index test	Time period	Age, mean (range)	Male gender (%)	Biopsy lesions (n)	Tumor lesions (n)
**NBI vs WLC**								
Shadpour et al.2016^28^	Unicentre, Iran	50	NBI	2012-2013	63.86 ± 10.05	34(68.0)	NR	95
Song et al.2016^26^	Unicentre, Korea	63	NBI	2012-2013	66(56-76)	39(61.9)	66	21
Kobotake et al.2015^34^	Unicentre, Japan	135	NBI	2010-2014	75	110(81.5)	383	120
Ye et al.2015^17^	Multicentre (8), China	384	NBI	NR	61(21-79)	267(69.5)	300	167
Shen et al.2012^27^	Unicentre, China	78	NBI	2009-2010	68 (33-75)	62(79.5)	309	211
Zhu et al. 2012^23^	Unicentre, China	12	NBI	2009-2010	57(28-73)	9(75.0)	31	9
Tatsugami et al.2010^25^	Unicentre, Japan	104	NBI	2007-2009	70.6(38-90)	88(84.6)	313	110
Cauberg et al.2009^46^	Multicentre (2), The Netherlands; Czech Republic	95	NBI	2007-2009	70.6(38.1-90.2)	70(73.7)	389	226
Herr et al.2008^37^	Unicentre, USA	427	NBI	2007	65 (26-90)	316(74.0)	NR	NR
**HAL vs WLC**								
Palou et al.2014^32^	Multicentre (8), Spain	283	HAL	2008-2009	67.5(42-95)	242(85.5)	1569	621
Lapini et al.2012^33^	Multicentre (5), Italy	96	HAL	2010-2011	NR	80(83.3)	234	108
Burgues et al.2011^55^	Multicentre (7), Spain	305	HAL	2006-2009	66.9(39-93)	270(88.5)	1659	600
Ray et al.2010^31^	Unicentre, Uk	27	HAL	2005-2006	70(49-82)	21(77.8)	120	NR
Schmidbauer et al.2009^29^	Unicentre, Austria	66	HAL	NR	63(38-84)	49(74.2)	364	NR
Geavlete et al.2008^39^	Unicentre, Romania	128	HAL	2007-2008	65(36-81)	NR	NR	NR
Fradet et al.2006^40^	Multicentre (18), USA, Canada And Europe	298	HAL	NR	67±11	223(74.8)	NR	113
Jichlinski et al.2003^35^	Multicentre (4), Swiss, Germany	52	HAL	2000-2001	72±12	38(73.1)	NR	143
**5-ALA vs WLC**								
Grimbergen et al.2003^10^	Unicentre, Netherlands	160	5-ALA	1998-2002	67(30-91)	NR	917	390
Filbeck et al.2002^42^	Unicentre, Germany	279	5-ALA	1997-2000	34-89	NR	636	336
Dominicis et al.2001^44^	Unicentre, Italy	49	5-ALA	NR	60(31-77)	42(85.7)	179	52
Ehsan et al.2001^43^	Unicentre, Germany	30	5-ALA	NR	55-89	19(63.3)	151	NR
Jeon et al.2001^36^	Unicentre, Korea	62	5-ALA	1997-1999	61.9(32-80)	57(91.1)	274	148
Zaak et al.2001^24^	Unicentre, Germany	605	5-ALA	NR	65.6(16-99)	472(78.0)	2475	552
Filbeck et al.1999^41^	Unicentre, Germany	123	5-ALA	1997	64.5(28-86)	NR	347	124
Riedl et al.1999^30^	Unicentre, Austria	52	5-ALA	NR	44-79	NR	53	123
D'hallewin et al.1998^45^	Unicentre, Belgium	16	5-ALA	NR	NR	NR	113	50

WLC: white light cystoscopy; NT: new technology; 5-ALA: 5-aminolaevulinic acid; HAL: hexylaminolevulinate; NBI: narrow band imaging; NR: not reported.

**Table 2:** Summary of variables of the included studies for sensitivity analysis.

Study	NMIBC (%)	Primary (%)
**NBI vs WLC**		
Shadpour et al.2016^28^	100	NR
Song et al.2016^26^	94.1	63.0
Kobotake et al.2015^34^	100	42.3
Ye et al.2015^17^	100	70.3
Shen et al.2012^27^	100	NR
Zhu et al. 2012^23^	100	42.0
Tatsugami et al.2010^25^	NR	NR
Cauberg et al.2009^46^	NR	35.9
Herr et al.2008^37^	100	0
**HAL vs WLC**		
Palou et al.2014^32^	94.1	67.1
Lapini et al.2012^33^	NR	36.5
Burgues et al.2011^55^	100	NR
Ray et al.2010^31^	100	0
Schmidbauer et al.2009^29^	93.1	NR
Geavlete et al.2008^39^	92.2	NR
Fradet et al.2006^40^	100	32
Jichlinski et al.2003^35^	100	NR
**5-ALA vs WLC**		
Grimbergen et al.2003^10^	90.0%	0
Filbeck et al.2002^42^	90.3%	NR
Dominicis et al.2001^44^	100	34.7
Ehsan et al.2001^43^	NR	NR
Jeon at al.2001^36^	NR	NR
Zaak et al.2001^24^	NR	NR
Filbeck et al.1999^41^	91.9	NR
Riedl et al.1999^30^	100	NR
D'hallewin et al.1998^45^	100	NR

NMIBC: non-muscle-invasive bladder cancer; WLC: white light cystoscopy; 5-ALA: 5-aminolaevulinic acid; HAL: hexylaminolevulinate; NBI: narrow band imaging; NR: not reported.

**Table 3:** Results of DTA analysis for all included studies

Study ID	Patient-level analysis								Lesion-level analysis						
	Pt No.	SSY	SPY	FPR	FNR	PPV	NPV		Ln No.	SSY	SPY	FPR	FNR	PPV	NPV
**NBI vs WLC**															
Shadpour et al.2016 28	50	NR	NR	NR	NR	NR	NR		175	69/80	70/85	15/85	11/80	69/84	74/75
Song et al.2016^26^	63	16/16	46/47	1/47	0/16	16/17	23/23		66	19/19	45/47	2/47	0/19	19/21	7/7
Kobotake et al.2015 34	135	NR	NR	NR	NR	NR	NR		379	78/84	227/263	36/263	6/84	78/114	203/203
Ye et al.2015^17^	103	56/56	16/45	29/46	0/56	56/85	8/8		300	124/126	92/133	41/133	2/126	124/165	83/85
Shen et al.2012^27^	78	47/47	9/22	13/22	0/47	47/47	7/7		309	160/160	98/134	36/134	0/160	160/196	72/72
Zhu et al. 2012^23^	12	NR	NR	NR	NR	NR	NR		31	4/6	19/22	3/22	2/6	4/7	20/20
Tatsugami et al. 2010 25	104	NR	NR	NR	NR	NR	NR		313	55/63	156/203	47/203	8/63	55/102	144/144
Cauberg et al.2009^46^	95	NR	NR	NR	NR	NR	NR		389	167/179	116/163	47/163	12/179	167/214	47/51
Herr et al.2008^37^	427	90/90	311/324	13/324	0/90	90/103	265/265		NR	NR	NR	NR	NR	NR	NR
**HAL vs WLC**															
Palou et al.2014^32^	283	NR	NR	NR	NR	NR	NR		1492	379/416	820/948	128/948	37/416	379/507	699/702
Lapini et al.2012^33^	96	NR	NR	NR	NR	NR	NR		234	82/83	101/126	25/126	1/83	82/107	80/81
Burgues et al.2011 55	305	NR	NR	NR	NR	NR	NR		1659	404/441	900/1059	159/1059	7/441	404/563	863/863
Ray et al.2010^31^	27	NR	NR	NR	NR	NR	NR		120	21/21	84/94	10/94	0/21	21/31	35/35
Schmidbauer et al.2009^29^	66	52/52	2/8	6/8	0/52	52/58	3/3		364	109/113	151/201	50/201	4/113	109/159	158/158
Geavlete et al.2008 39	128	NR	NR	NR	NR	NR	NR		243	87/93	56/103	47/103	6/93	87/134	76/82
Fradet et al.2006^40^	196	40/48	128/138	10/138	8/48	40/50	106/113		206	77/83	101/112	11/112	6/83	77/88	63/71
Jichlinski et al.2003^35^	52	33/33	7/17	10/17	0/33	33/43	3/3		143	205/254	269/343	74/343	49/254	205/279	306/314
**5-ALA vs WLC**															
Grimbergen et al.2003 10	160	NR	NR	NR	NR	NR	NR		889	232/244	409/527	118/527	12/244	232/350	248/257
Filbeck et al.2002 42	279	168/168	93/102	9/102	0/168	168/177	81/81		NR	NR	NR	NR	NR	NR	NR
Dominicis et al.2001 44	49	NR	NR	NR	NR	NR	NR		179	2/9	84/127	43/127	7/9	2/45	80/80
Ehsan et al.2001^43^	30	NR	NR	NR	NR	NR	NR		151	39/40	71/91	20/91	1/40	39/59	59/59
Jeon at al.2001^36^	62	NR	NR	NR	NR	NR	NR		257	71/74	69/126	57/126	3/74	71/128	54/54
Zaak et al.2001^24^	605	288/363	271/460	189/460	75/363	288/477	55/108		NR	NR	NR	NR	NR	NR	NR
Filbeck et al.1999 41	123	NR	NR	NR	NR	NR	NR		341	75/80	185/223	38/223	5/80	75/113	78/78
Riedl et al.1999^30^	52	26/26	10/18	8/18	0/26	26/34	6/6		NR	NR	NR	NR	NR	NR	NR
D'Hallewin et al. 1998 45	16	NR	NR	NR	NR	NR	NR		113	11/14	27/63	36/63	3/14	11/47	34/34

NMIBC: non-muscle-invasive bladder cancer; Pt: patients; Ln: lesions; WLC: white light cystoscopy; 5-ALA: 5-aminolaevulinic acid; HAL: hexylaminolevulinate; NBI: narrow band imaging; NT: new technology; SSY: sensitivity; SPY: specificity; FPR: false positive rate; FNR: false negative rate; PPV: positive predictive value; NPV: negative predictive value; NR: not reported.

**Table 4:** Summary of results of DTA analysis for index tests

Study ID	Patient-level analysis				Lesion-level analysis		
	Median	Lower Quartile	Upper Quartile		Median	Lower Quartile	Upper Quartile
**NBI vs WLC (n=4)**					**NBI vs WLC (n=8)**		
Sensitivity	100	100	100		93.08	87.04	98.81
Specificity	68.45	39.57	96.47		79.60	72.64	86.32
Positive predictive value	90.75	82	95.59		76.59	65.60	81.76
Negative predictive value	100	100	100		100	98.41	100
False positive rate	31.55	3.54	60.08		20.40	13.68	27.36
False negative rate	0	0	0		6.92	1.19	12.96
**HAL vs WLC (n=3)**					**HAL vs WLC (n=8)**		
Sensitivity	100	91.67	100		93.16	91.48	97.04
Specificity	41.18	33.09	66.97		82.57	77.60	87.21
Positive predictive value	80.00	78.37	84.83		72.62	68.35	75.22
Negative predictive value	100	96.90	100		99.17	96.26	100
False positive rate	58.82	33.03	66.91		17.43	12.79	22.40
False negative rate	8.33	4.17	12.50		5	1.49	7.65
**5-ALA vs WLC (n=3)**					**5-ALA vs WLC (n=6)**		
Sensitivity	100	89.67	100		94.42	82.37	95.73
Specificity	58.91	57.23	75.04		71.88	57.61	77.92
Positive predictive value	76.47	68.42	85.69		60.79	31.42	66.24
Negative predictive value	100	75.46	100		100	100	100
False positive rate	41.09	24.96	42.77		28.12	22.08	42.39
False negative rate	0	0	10.33		5.58	4.27	17.63

NMIBC: non-muscle-invasive bladder cancer; WLC: white light cystoscopy; 5-ALA: 5-aminolaevulinic acid; HAL: hexylaminolevulinate; NBI: narrow band imaging; NR: not reported.

**Table 5:** Sensitivity analysis of studies with low to moderate RoB for index tests

Study ID	Patient-level analysis				Lesion-level analysis		
	Median	Lower Quartile	Upper Quartile		Median	Lower Quartile	Upper Quartile
**NBI vs WLC (n=3)**					**NBI vs WLC (n=6)**		
Sensitivity	100	100	100		95.85	88.80	99.60
Specificity	40.91	38.24	69.39		74.99	71.66	80.98
Positive predictive value	94.12	80.0	97.06		79.84	75.87	82.02
Negative predictive value	100	100	100		99.33	97.90	100
False positive rate	59.09	30.61	61.07		25.01	19.02	28.34
False negative rate	0	0	0		4.15	0.40	11.20
**HAL vs WLC (n=1)**					**HAL vs WLC (n=6)**		
Sensitivity	83.33	-	-		95.00	92.97	98.21
Specificity	92.75	-	-		83.33	76.38	88.65
Positive predictive value	80.00	-	-		71.65	67.94	76.16
Negative predictive value	93.81	-	-		99.17	94.20	99.89
False positive rate	7.25	-	-		16.67	11.35	23.62
False negative rate	16.67	-	-		5.00	1.79	7.03
**5-ALA vs WLC (n=1)**					**5-ALA vs WLC (n=4)**		
Sensitivity	100	-	-		95.51	94.75	96.33
Specificity	91.18	-	-		77.82	71.90	79.26
Positive predictive value	94.92	-	-		66.19	63.44	66.31
Negative predictive value	100	-	-		100	99.12	100
False positive rate	8.82	-	-		22.18	20.74	28.10
False negative rate	0	-	-		4.49	3.67	5.25

NMIBC: non-muscle-invasive bladder cancer; WLC: white light cystoscopy; 5-ALA: 5-aminolaevulinic acid; HAL: hexylaminolevulinate; NBI: narrow band imaging; NR: not reported.

**Table 6:** Sensitivity analysis of studies with more than 100 patients on Patient-level analysis and Lesion-level analysis

Study ID	Patient-level analysis					Lesion-level analysis		
	Median	Lower Quartile	Upper Quartile			Median	Lower Quartile	Upper Quartile
NBI vs WLC (n=2)						NBI vs WLC (n=3)		
Sensitivity	100	-	-			92.86	90.08	95.63
Specificity	65.78	-	-			76.85	73.01	81.58
Positive predictive value	76.63	-	-			68.42	61.17	71.79
Negative predictive value	100	-	-			100	98.82	100
False positive rate	33.53	-	-			23.15	18.42	26.99
False negative rate	0	-	-			7.14	4.37	9.92
**HAL vs WLC (n=1)**						**HAL vs WLC (n=4)**		
Sensitivity	83.33	-	-			92.19	91.48	92.97
Specificity	92.75	-	-			85.74	77.33	87.42
Positive predictive value	80	-	-			73.26	70.05	77.94
Negative predictive value	93.81	-	-			96.13	91.70	99.68
False positive rate	7.25	-	-			14.26	12.58	22.67
False negative rate	16.67	-	-			6.84	5.24	7.65
**5-ALA vs WLC (n=2)**						**5-ALA vs WLC (n=2)**		
Sensitivity	89.67	-	-			94.42	-	-
Specificity	75.05	-	-			80.28	-	-
Positive predictive value	77.65	-	-			66.33	-	-
Negative predictive value	75.47	-	-			98.25	-	-
False positive rate	24.96	-	-			19.72	-	-
False negative rate	10.33	-	-			5.58	-	-
								

NMIBC: non-muscle-invasive bladder cancer; WLC: white light cystoscopy; 5-ALA: 5-aminolaevulinic acid; HAL: hexylaminolevulinate; NBI: narrow band imaging; NR: not reported.
