# Supplementary material for: Diagnostic accuracy of photodynamic diagnosis with 5-aminolevulinic acid, hexaminolevulinate and narrow band imaging for non-muscle invasive bladder cancer
Source: J Cancer. 2020 Jan 1;11(5):1082–93. doi: 10.7150/jca.34527 (PMC6959070; doi:10.7150/jca.34527)
Supplement: Supplementary file 1 — Supplementary figures and tables. [file jcav11p1082s1.pdf]

Supplementary figures

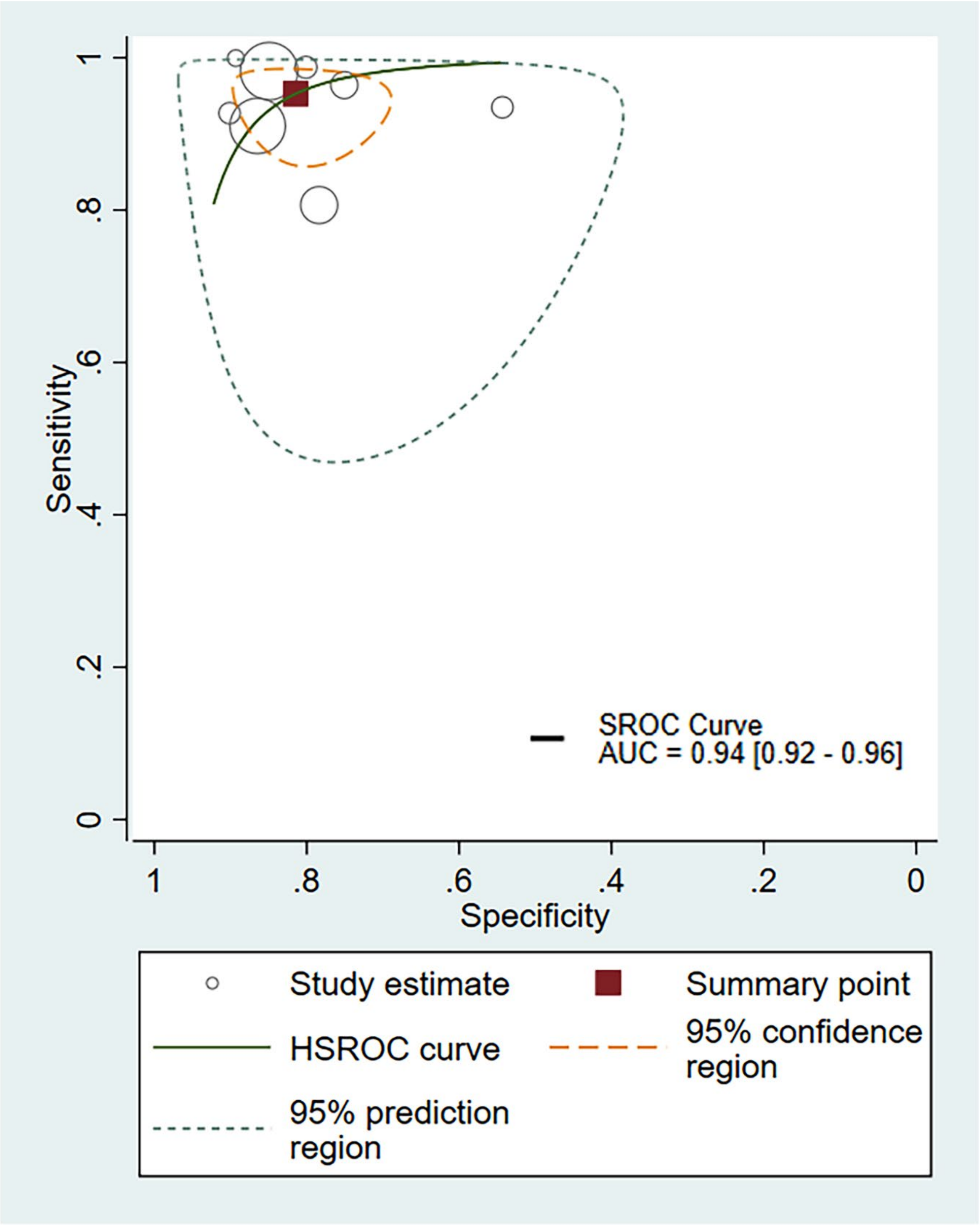

Supplementary figure 1. The HSROC curve for HAL diagnosing NMIBC comparing with WLC in lesion level.

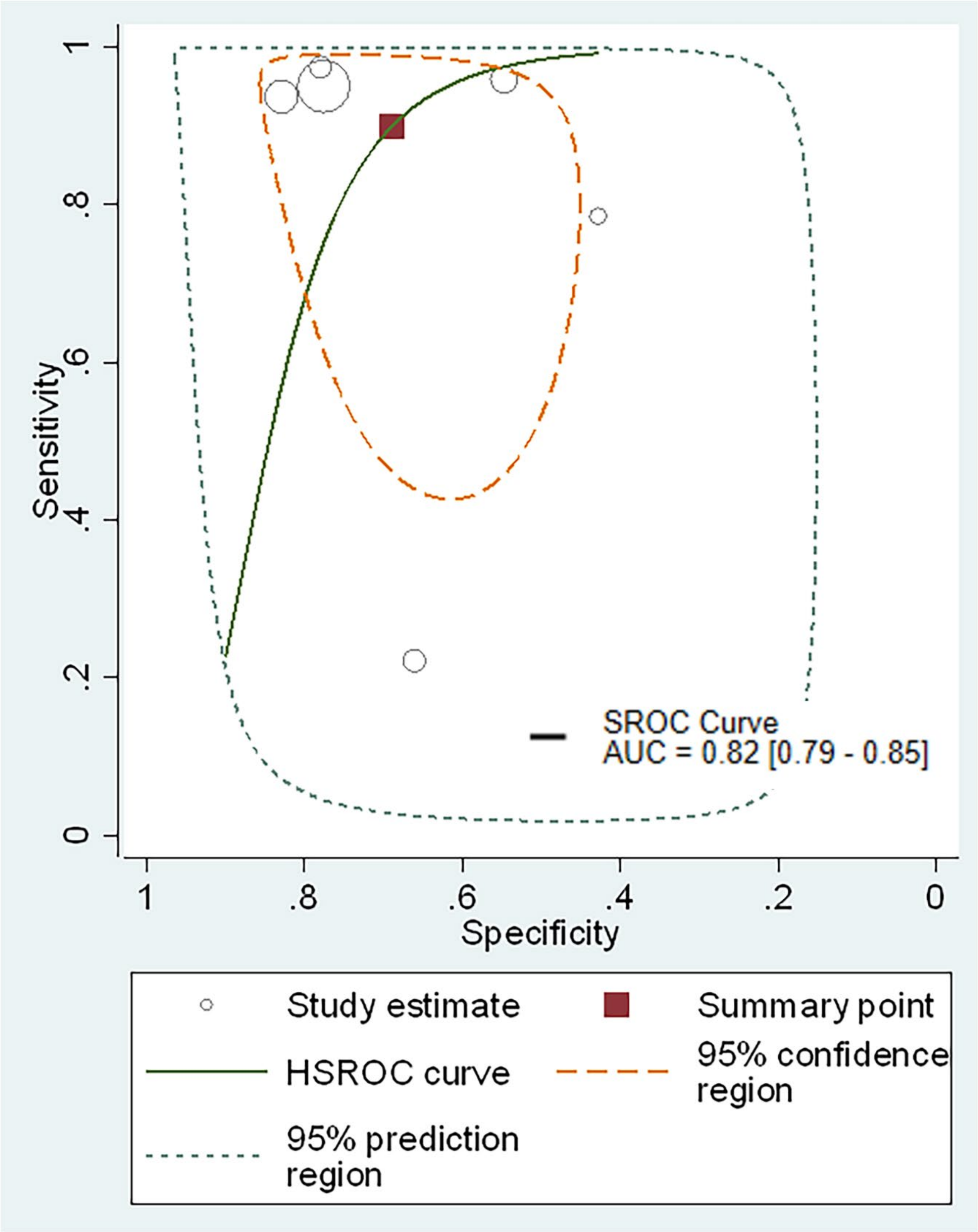

Supplementary figure 2. The HSROC curve for 5-ALA diagnosing NMIBC comparing with WLC in lesion level.

Supplementary tables

Supplementary Table 1: Sensitivity analysis of DTA for NBI (6 studies) with low to moderate RoB.

	Sensitivity	Specificity	PPV	NPV	FP	FN
**Median (IQR)**	95.85% (88.80-99.60%)	74.99% (71.66-80.98%)	79.84% (75.87-82.02%)	99.33% (97.90-100%)	25.01% (19.02-28.34%)	4.15% (0.40-11.20%)
**Minimum**	86.25%	69.17%	53.92%	92.16%	4.26%	0%
**Maximum**	100%	95.74%	90.48%	100%	30.83%	13.75%

Supplementary Table 2: Sensitivity analysis of DTA for HAL (6 studies) with low to moderate RoB.

	Sensitivity	Specificity	PPV	NPV	FP	FN
**Median (IQR)**	95.00% (92.97-98.21%)	83.33% (76.38-88.65%)	71.65% (67.94-76.16%)	99.17% (94.20-99.89%)	16.67% (11.35-23.62%)	5.00% (1.79-7.03%)
**Minimum**	91.11%	54.37%	64.93%	88.73%	9.82%	0%
**Maximum**	100%	90.18%	87.5%	100%	45.63%	8.89%

Supplementary Table 3: Sensitivity analysis of DTA for 5-ALA (4 studies) with low to moderate RoB.

	Sensitivity	Specificity	PPV	NPV	FP	FN
**Median (IQR)**	95.51% (94.75-96.33%)	77.82% (71.90-79.26%)	66.19% (63.44-66.31%)	100% (99.12-100%)	22.18% (20.74-28.10%)	4.49% (3.67-5.25%)
**Minimum**	93.75%	54.76%	55.47%	96.50%	17.04%	2.50%
**Maximum**	97.50%	82.96%	66.37%	100%	45.24%	6.25%
